# N-Butanol Extract of Modified You-Gui-Yin Attenuates Osteoclastogenesis and Ameliorates Osteoporosis by Inhibiting RANKL-Mediated NF-κB Signaling

**DOI:** 10.3389/fendo.2022.925848

**Published:** 2022-06-24

**Authors:** Qinghe Zeng, Rui Xu, Houfu Ling, Shan Zhao, Xu Wang, Wenhua Yuan, Mancang Gu, Taotao Xu, Pinger Wang, Hongfeng Ruan, Hongting Jin, Hangbo Qu, Fusheng Ye, Jiali Chen

**Affiliations:** ^1^ Institute of Orthopedics and Traumatology, The First Affiliated Hospital of Zhejiang Chinese Medical University, Hangzhou, China; ^2^ The First College of Clinical Medicine, Zhejiang Chinese Medical University, Hangzhou, China; ^3^ Department of Orthopaedics, Affiliated Hospital of Jiangxi University of Traditional Chinese Medicine, Nanchang, China; ^4^ Department of Orthopaedics, The First Affiliated Hospital of Zhejiang Chinese Medical University, Hangzhou, China; ^5^ The College of Pharmaceutical Sciences, Zhejiang Chinese Medical University, Hangzhou, China; ^6^ Department of Orthopaedic Surgery, Zhejiang Hospital, Hangzhou, China; ^7^ Affiliated Xiaoshan Hospital, Hangzhou Normal University, Hangzhou, China

**Keywords:** N-butanol extraction, Modified You-Gui-Yin, postmenopausal osteoporosis, osteoclast formation, NF-κB pathway

## Abstract

Postmenopausal Osteoporosis (PMOP) is the most prevalent primary osteoporosis, attributable to an imbalance in osteoblast and osteoclast activity. Modified You-Gui-Yin (MYGY), a traditional Chinese herbal formula, is able to effectively treat PMOP, while the critical components and pharmacological mechanisms of MYGY are still unclear. In this study, we aimed to investigate the therapeutic effects and underlying mechanisms of N-butanol extract of MYGY (MYGY-Nb) in ovariectomized (OVX)-induced osteoporosis mice. Histological staining and micro-computed tomography (μCT) analysis showed that MYGY-Nb was more effective in the suppression of OVX-induced bone loss than MYGY original formula. Subsequently, liquid chromatography and mass spectrometry analysis identified 16 critical compounds of MYGY-Nb and some of them are reported to affect osteoclast functions. Furthermore, *in vivo* and *in vitro* experiments demonstrated that MYGY-Nb significantly attenuated osteoclastogenesis by down-regulating RANKL-mediated NF-κB signaling. In conclusion, our study indicated that MYGY-Nb suppresses NF-κB signaling and osteoclast formation to mitigate bone loss in PMOP, implying that MYGY-Nb and its compounds are potential candidates for development of anti-PMOP drugs.

## Introduction

Osteoporosis is a skeletal disease characterized by reduced bone mass density and strength, increased skeletal fragility and fractures (1). As the population ages, the worldwide patients caused by osteoporosis might well reach 221 million by 2050 (2–4), and the medical and socioeconomic burden of osteoporosis, particularly postmenopausal osteoporosis (PMOP), will increase further (5). PMOP, mainly contributed by menopause – the cessation of ovarian function, is the most common primary osteoporosis. However, due to the cost and side effects of available drugs, most cases of PMOP have not been effectively treated so far (6). Therefore, the development of novel effective therapeutic agents with minimal side effects for the PMOP treatment is urgently needed (7).

Traditional Chinese medicine (TCM) has been used for thousands of years to treat various chronic diseases, especially osteoporosis (8–11). The Modified You-Gui-Yin (MYGY) is a well-known TCM formula, which is derived from You-Gui-Yin classic prescription recorded in *Jing Yue Quan Shu* written by Jingyue Zhang in the Ming Dynasty. The MYGY consists of ten herbs: Persicae semen, Cinnamomi cortex, Monkshood root, Eucommiae cortex, Lycii fructus, Dioscoreae rhizome, Carthami flos, Corni fructus, Glycyrrhizae radix and Preparata rehmanniae radix (**Table 1**), and has been widely applied to treat various chronic diseases with fewer side effects (12, 13). Our recent study has shown that MYGY-based therapies achieve significant clinical outcomes in PMOP management (14). In addition, several herbs of MYGY have been reported to ameliorate osteoporosis by inhibiting osteoclast activity, such as Glycyrrhizae radix, Dioscoreae rhizoma, Corni fructus (12, 15, 16). However, the underlying mechanisms and functional compounds of MYGY in the treatment of PMOP have not been fully elucidated, which limits the further development and application of MYGY.

**Table 1:** The herbal components of MYGY.

Chinese name	Botanical name	Common name	Parts used	Proportion
Tao ren	*Prunus persica* (L.) Batsch	Persicae semen	Seed	11.8%
Rou gui	*Cinnamomum cassia* Presl	Cinnamomi cortex	Bark	5.9%
Fu zi	*Aconitum carmichaeli* Debx.	Monkshood root	Root	11.8%
Du zhong	*Eucommia ulmoides* Oliv.	Eucommiae cortex	Bark	11.8%
Gou qi	*Lycium barbarum* L.	Lycii fructus	Fruit	11.8%
Shan yao	*Dioscorea opposita* Thunb.	Dioscoreae rhizoma	Root and rhizome	11.8%
Hong hua	*Carthamus tinctorius* (L.)	Carthami flos	Flower	5.9%
Shan zhu yu	*Cornus officinalis* Sieb. et Zucc.	Corni fructus	Fruit	5.9%
Gan cao	*Glycyrrhiza uralensis* Fisch.	Glycyrrhizae radix	Root	5.9%
Shu di huang	*Rehmannia glutinosa* Libosch.	Preparata rehmanniae radix	Root	17.4%

In the process of bone reconstruction, the cumulative imbalance in the activity of osteoblasts and osteoclasts can lead to a decrease in bone mass, which is fundamental to the pathogenesis of PMOP (17). Of note, this imbalance is mainly due to the osteoclast reabsorption of bone in excess of osteoblasts production of mineralized extracellular matrix (18). Osteoclasts are produced by the differentiation of precursors from a monocyte/macrophage lineage that is derived from bone marrow hematopoietic stem cells (19). Thus, the prevention of these stem cells from differentiation of osteoclast is one of the most important strategies in PMOP treatment.

The survival, proliferation, differentiation and activation of osteoclasts require the presence of RANKL (receptor activator of nuclear factor kappa B ligand) and M-CSF (macrophage colony-stimulating factor) (20). In the process of osteoclastogenesis, RANKL interacts with RANK (receptor of RANKL) to induce the activation of NF-κB through the recruitment of adapter molecules such as TRAF6 (TNF receptor-associated factor 6), the degradation of IκB (the suppressor of NF-κB), and the phosphorylation of p65 (the subunit of NF-κB). These signaling cascades subsequently activate NFATc1 (nuclear factor of activated T cells, cytoplasmic 1), known as the primary transcription factor for osteoclastogenesis (21, 22). During the postmenopausal period, the expression of various inflammation inhibitors was found to decrease, but RANKL increased significantly, suggesting a critical role for RANKL in PMOP (23). And NF-κB signaling pathway participates in regulating the proliferation and function of osteoclasts (24–27). Thus, the suppression of osteoclast formation by targeting the RANKL-mediated NF-κB signaling pathway is probably a potential treatment strategy for osteoclastogenesis-related disorders, especially for PMOP.

In this study, we investigated the impact of N-butanol extract of MYGY (MYGY-Nb) on OVX mice and primary bone marrow-derived monocytes (BMMs). We found that MYGY-Nb possessed a stronger anti-osteoporosis effect with attenuating bone loss and osteoclast formation. Moreover, the mechanism by which MYGY-Nb suppressed osteoclastogenesis might be that MYGY-Nb inhibited RANKL-mediated NF-κB signaling. Taken together, our findings implicated that MYGY-Nb might be an effective medicine for treating PMOP.

## Materials and Methods

### Preparation of MYGY-Nb

All herbs of MYGY (**Table 1**) were provided by the First Affiliated Hospital of Zhejiang Chinese Medical University (Hangzhou, China). The N-butanol extract of MYGY was prepared as follows: Rou gui and Tao ren were mixed in a ratio of 1:2, soaked in 60% ethanol for 1 h, and then extracted 3 times by reflux method for 1.5 hours each. Other eight herbs including Fu zi, Du zhong, Gou qi, Shan yao, Hong hua, Shan zhu yu, Gan cao and Shu di huang were mixed in a1:3:2:2:2:1:2:1 ratio, steeped in 12 volumes of distilled water for 1 hour, and then extracted 3 times for 1.5 h each. Afterwards, the above two extracts were mixed and concentrated into MYGY extractum (3.4 g crude drug/mL). The above procedure is the preparation method of MYGY concentrate. After that, to extract its optimal anti-osteoporosis fraction, 95 ml MYGY extractum was dissolved into 300 ml distilled water, then, 300 ml N-butanol was mixed in equal proportion. After overnight emulsification and precipitation, the supernatant was collected and again extracted by the above method 3 times. Then, the supernatant was concentrated under reduced pressure until the N-butanol was completely evaporated to obtain the crude extract powder, namely MYGY-Nb. The MYGY-Nb powder was dissolved in 300ml sterile distilled water and stored at -20°C.

### Ultra-High-Performance Liquid Chromatography Coupled With Quadrupole Time-of-Flight Tandem Mass Spectrometry (UHPLC-Q-TOF-MS) Analysis

The MYGY-Nb powder was dissolved in methanol to 1 μg/mL for liquid chromatography analysis. Then a Waters Acquity UPLC BEH C_18_ column (100 mm×2.1 mm, 1.7 μm) was used to separate the compounds in MYGY-Nb with the mobile phase of acetonitrile (A)-0. 1% formic acid water (B), and the elution was gradient elution (0~7 min, 95% B; 7~15 min, 85% B; 15~18 min, 65% B; 18~23 min, 40% B; 23~27 min, 10% B; 27~29 min, 95% B) with volume flow rate of 0.3 mL/min, column temperature of 40°C and injection volume of 5 μL.

The MS^E^ continuum mode of the SYNAPT G2-Si mass spectrometer was used to detect constituents of MYGY-Nb in the scan range of m/z 50~1500. In the ESI^-^/ESI^+^ modes, the capillary and cone well voltages were 3.0 kV and 40 V, respectively, and the argon flow rate was 0.15 mL/min. The cone well gas flow rate was 50 L/h at 120°C, and the desolvation flow rate was 800 L/h at 400°C. The 100 ng/mL solution of leucine-enkephalin (ESI^-^: m/z 555.261 5, ESI^+^: m/z 556.2771) was used as a calibration standard (Lock Spray TM) with a volume flow rate of 15 μL/min, and the instrument was calibrated with sodium formate for accurate mass determination. The UNIFI V1.8 software was used for data analysis.

### Animal Grouping and Drug Administration

10-week-old female C57BL/6J mice, supplied by Zhejiang Chinese Medical University Animal Center (Hangzhou, China), were maintained under standard feeding conditions with fresh water and food freely available. All mouse experiments were conducted in the pathogen-free laboratory of the Animal Center of Zhejiang Chinese Medical University (Hangzhou, China). Animals were randomly assigned into four groups (n = 6 in each group): (1) sham group, subjected to sham operation, (2) ovariectomy group (OVX), subjected to bilateral ovariectomy, (3) OVX+MYGY-Nb group, subjected to bilateral ovariectomy and then orally administrated MYGY-Nb extraction (20 mL/kg/day) for 12 weeks. (4) OVX+MYGY group, subjected to bilateral ovariectomy and then orally administrated MYGY extraction (20 mL/kg/day) for 12 weeks. The mice in other groups were addressed with the same dose of physiologic saline.

### Micro-Computed Tomography (μCT) Analysis

After the last administration, mice were sacrificed by pentobarbital sodium overdose, and then their femurs were isolated for μCT scanning (Skyscan1176, Belgium) at a 10 µm resolution. The three-dimensional (3D) structure reconstruction of the distal femurs was performed using the NRecon software. The bone mineral density (BMD), trabecular separation (Tb.Sp, mm), trabecular number (Tb.N, 1/mm), trabecular thickness (Tb.Th, mm) and bone volume fraction (BV/TV, %) were analyzed as described previously (28).

### Immunohistochemistry (IHC) and Histology

After μCT analysis, it was processed into 3-μm-thick paraffin sections of the femur as previously described (28). Subsequently, the sections were stained by Alcian blue hematoxylin/Orange G(ABH) for morphological analysis. The trabecular area (%) and lipid droplet area (%) of the region of the distal femur were calculated by OsteoMetrics software (Decatur, GA, USA). In IHC assays. the sections were incubated with 0.3% Triton X-100 for 10 min at room temperature and then treated with endogenous peroxidase blocker (ZSGB-BIO, PV-6001, Beijing, China) for 20 min. Subsequently, sections were incubated with primary antibodies of alkaline phosphatase (ALP, Arigo, ARG57422, 1:200), IKB alpha (IKBα, HuaBio, ET1603-6, 1:200), phosphorylated-p65(p-p65, Arigo, ARG51518,1:300), Nuclear factor of activated T-cells, cytoplasmic 1 (NFATc1, Novusbio, NB300-620, 1:300), and Receptor activator nuclear factor-κB (NF-κB) ligand (RANKL, Abcam, Ab45039, 1:500) overnight at 4°C, respectively. The next day, these sections were treated with a homologous secondary antibody for 20 min. The diaminobenzidine (DAB) solution (ZSGB-BIO, ZLI-9018, Beijing, China) was used to detect positive staining followed by hematoxylin re-staining. The quantification of positive staining was analyzed by using the software of Image-Pro Plus (Media Cybernetics, Silver Spring, USA).

### Cell Culture

Briefly, BMMs were separated from the femur and tibia of C57BL/6 J mice as previously described (29), and cultured in α minimum essential medium (α-MEM, Gibco, New York, USA) containing 10% fetal bovine serum (FBS, HyClone, Beijing, China), 1% streptomycin/penicillin (HyClone, Beijing, China) and 30 ng/mL M-CSF (R&D Systems, Minneapolis, MN, USA) in a humidified atmosphere at 37°C containing.

### Cell Viability Assays

Cell Counting Kit-8 (CCK-8, Bioss, Beijing, China) assay was used to examine the viability of cells. The BMMs were seeded into 96-well plates at a density of 1 × 10^4^ cells/well. After 24 h, BMMs were treated with 0, 10, and 100 μg/mL MYGY-Nb for 24, 48 or 72 h. Then the CCK8 solution was added to the culture medium and incubated at 37°C for 2 h. The absorbance was measured by BioTek Synergy HT spectrophotometer at absorbance was measured at 450 nm.

### Osteoclast Formation and Tartrate-Resistant Acid Phosphatase (TRAP) Staining

To examine osteoclast formation *in vitro*, BMMs were cultured for 6 days in 24-well plates (1 × 10^5^ cells/well) with α-MEM containing 10% FBS, 1% streptomycin/penicillin, 30 ng/mL M-CSF, 50 ng/mL RANKL, and 0 or 10 μg/mL MYGY-Nb. And then, the cells were stained using the TRAP staining kit (Sigma-Aldrich, St. Louis, MO, USA). Osteoclasts (cells with more than three nuclei) were quantified by using Image J software (NIH, Bethesda, Maryland, USA).

### Western Blotting Analysis

The BMMs were cultured with 30 ng/mL M-CSF, 50 ng/mL RANKL and MYGY-Nb (0 or 10 μg/mL) for 4 days, and then mixed with RIPA buffer to extract total protein, the concentration of which was determined by bicinchoninic acid (BCA). Proteins were separated on 10% SDS-PAGE gels (20 μg/lane) and then transferred to PVDF membranes. The membranes were blocked with 5% skim milk for 1 h, followed by incubating with primary antibodies (IKBα, 1:1000 dilution, Cell Signaling Technology), (p65, 1:1000 dilution, Cell Signaling Technology), (p-p65, 1:1000 dilution, Cell Signaling Technology), (NFATc1, 1:1000 dilution, Novus Biologicals) and (β-actin, 1:10,000 dilution, Sigma-Aldrich) overnight at 4°C. After incubation with the corresponding secondary antibodies, proteins on the membrane were detected by the ultra ECL reagent kit (4A Biotech, Beijing, China) on an Image Quant LAS 4000 (EG, United States). The grey value was calculated by the software of Image J.

### Statistical Analysis

All data were analyzed using GraphPad Prism and presented in mean ± S.D. One-way ANOVA and Student’s t-test were performed to analyze the difference between groups. A value of *P*<0.05 was considered statistically significant.

## Results

### MYGY-Nb Significantly Reduces Bone Loss in OVX Mice

**Figure 1:**
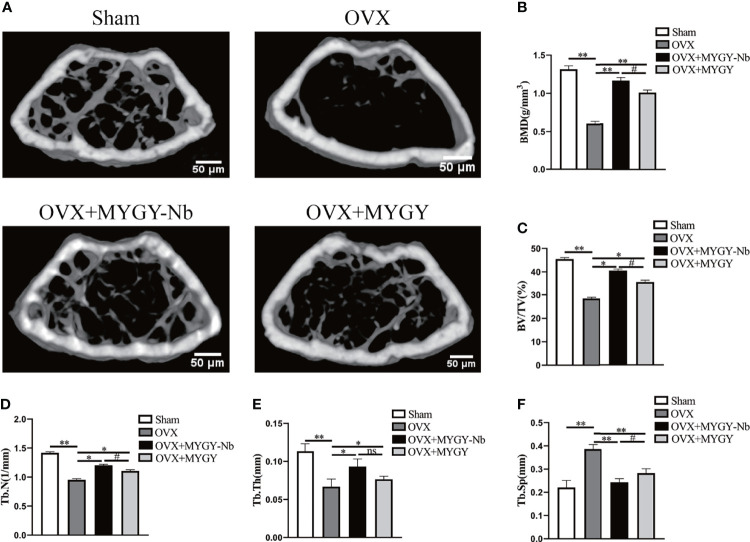
Changes of bone structure in OVX mice, MYGY original formula and MYGY-Nb administration mice. **(A)** Representative μCT images. Quantification of microstructural parameters including BMD **(B)**, BV/TV **(C)**, Tb. N **(D)**, Tb. Th **(E)** and Tb. Sp **(F)**. Scale bars: 50 μm. Data were presented as means ± S.D. *P < 0.05; **P < 0.01; ns: no significant difference; ^#^ *P* < 0.05. n = 6 in each group.

To determine the effect of MYGY-Nb on PMOP, C57BL/6J mice were subjected to OVX surgery and then treated with MYGY original formula and MYGY-Nb (20 mL/kg/day) respectively for 12 weeks without any adverse effects. The μCT results showed a significant reduction in bone mass and changes of bone microarchitecture in OVX mice compared to controls (**Figure 1A**). After administration with MYGY original formula and MYGY-Nb, these OVX-induced abnormal symptoms were effectively alleviated, as illustrated by the increases in BMD, Tb.N, Tb.Th and BV/TV, as well as the decline in Tb.Sp (**Figures 1B–F**). Of note, MYGY-Nb was more effective in inhibiting bone loss than MYGY original formula (**Figures 1A–F**).

**Figure 2:**
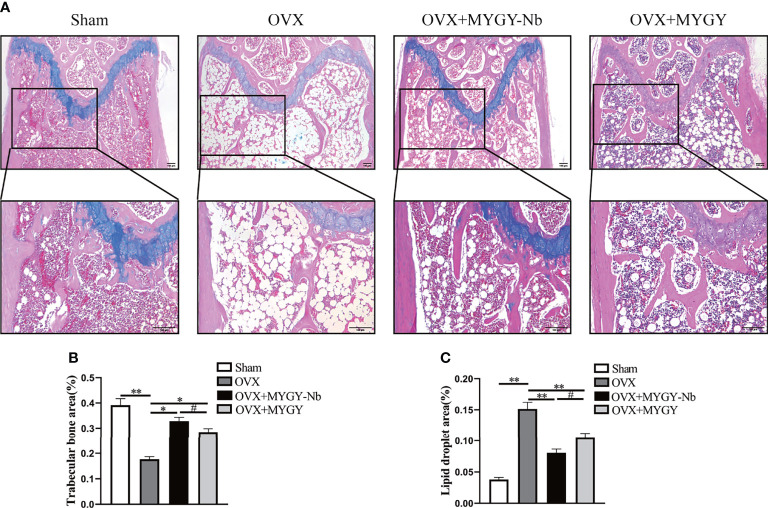
MYGY-Nb and MYGY original formula protect against bone loss in OVX mice. **(A)** Alcian Blue Hematoxylin/Orange G staining of distal femur. Scale bars: 100 μm. **(B)** The area of trabecular bone (%). **(C)** The area of lipid droplets (%). Data were presented as means ± S.D. *P < 0.05; **P < 0.01; ^#^ *P* < 0.05. n = 6 in each group.

Additionally, ABH staining revealed that compared with sham mice, OVX mice displayed sparse trabeculae and massive adiposity accumulation (**Figures 2A–C**). As expected, in both MYGY original formula and MYGY-Nb administration groups, these OVX-induced effects were greatly inhibited, and MYGY-Nb showed a significantly better performance than MYGY original formula (**Figures 2A–C**).

**Figure 3:**
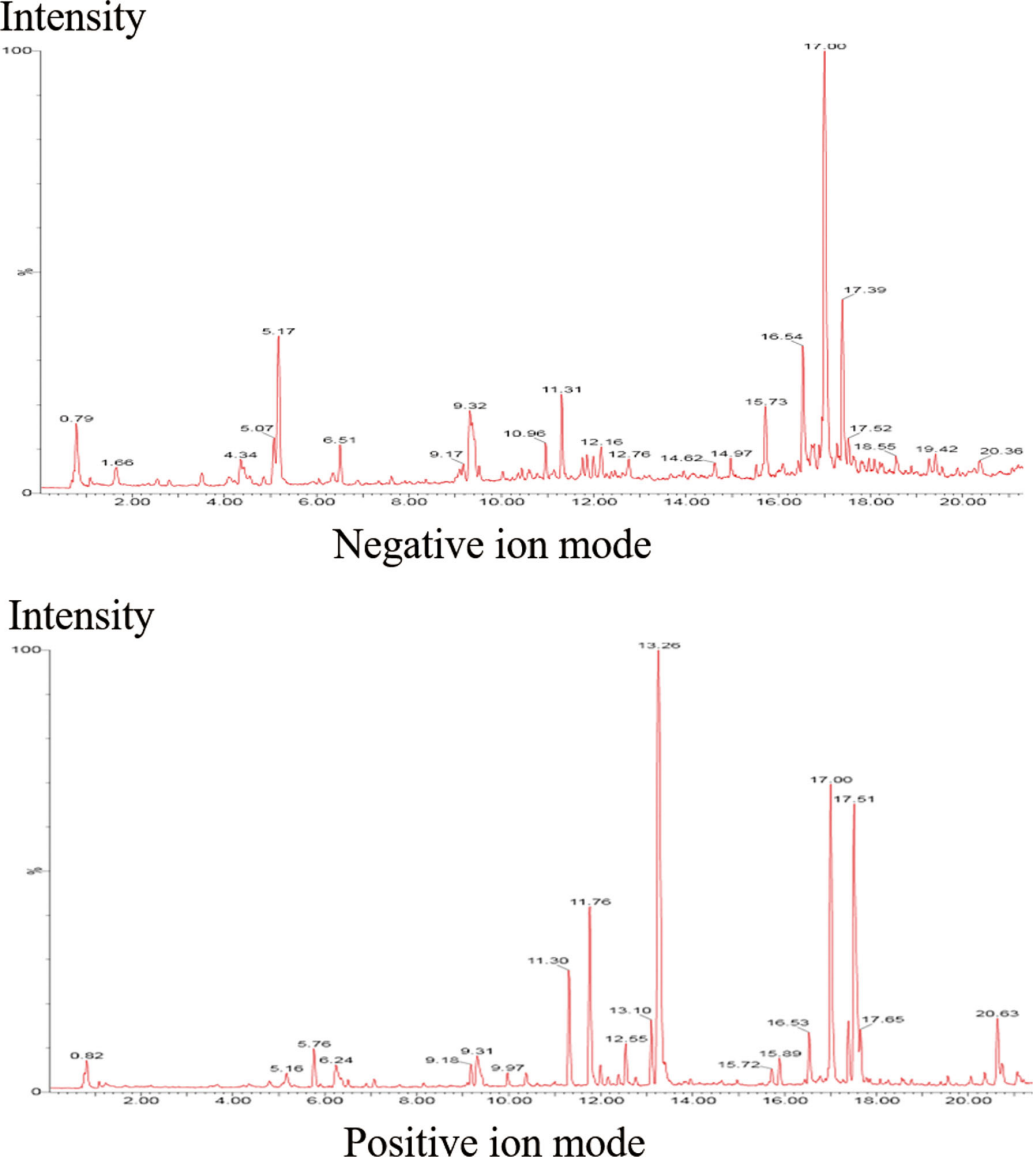
The total ion chromatogram of MYGY-Nb in Negative and Positive ion mode.

Therefore, we sought to identify the compounds of MYGY-Nb based on UHPLC-Q-TOF/MS analysis. **Figure 3** showed the total ion chromatograms of MYGY-Nb in negative and positive ion modes. By comparison with the reference standard, retention time and published literatures, 16 main chemical constituents in MYGY-Nb were identified preliminarily (**Table 2**), among which Kynnipin glycosides, Rutin (Rutinoside)_1, Glycyrrhizin and Glycyrrhizic acid have shown the inhibiting functions in osteoclast formation (30–33).

**Table 2:** The detailed information of compounds contained in MYGY-Nb.

Group Name	Chemical formula	Observation retention time (min)	Detector count	Response	Additives	Observed m/z	Measured molecular mass number (Da)	Molecular mass number (Da)
Raffinose	C18H32O16	0.48	139297	11541	+HCOO	539.1379	504.1685	504.16903
Gallic acid	C7H6O5	0.89	49696	46241	-H, +Cl	169.0140	170.0213	170.02152
Morroniside	C17H26O11	2.89	278503	155160	+HCOO, -H, +Cl	451.1456	406.1474	406.14751
Amygdalin	C20H27NO11	3.61	1561535	694807	+HCOO, -H, +Cl	502.1562	457.158	457.15841
Kynnipin glycosides	C17H24O10	4.77	284673	221943	+HCOO, -H, +Cl	433.1343	388.1361	388.13695
Glycyrrhizin	C21H22O9	7.25	490335	394007	-H, +Cl	417.1189	418.1261	418.12638
Rutin (Rutinoside)_1	C27H30O16	7.50	496688	375064	-H,	609.146	610.1532	610.15338
Cartormin	C27H29NO13	9.59	233960	24332	-H, +Cl	574.1557	575.163	575.16389
Liquiritin (glycyrrhetinin)	C21H22O9	10.51	130089	102027	-H, +Cl	417.1186	418.1259	418.12638
Glycyrrhiza chalcone B	C16H14O5	10.72	23045	18160	-H	285.0763	286.0836	286.08412
Hobutamine A	C32H45NO10	13.64	27073	16488	+HCOO, +Cl	648.3024	603.3042	603.30435
Formononetin	C16H12O4	14.32	78984	43437	-H	267.0658	268.0731	268.07356
Glycyrrhizolide	C30H44O4	15.26	122727	90243	-H	467.3311	468.3238	468.32396
Glycyrrhizic acid	C42H62O16	16.10	5535136	3306800	-H	821.3966	822.4038	822.40379
18β-Glycyrrhetinic acid	C30H46O4	16.60	365954	14457	-H	469.3466	470.3393	470.33961
Glycyrrhizic acid	C42H62O16	16.75	249884	163193	-H, +Cl	821.3959	822.4031	822.40379

### MYGY-Nb Inhibits the OVX-Induced Increase in Osteoclast and Decrease in Osteoblast

**Figure 4:**
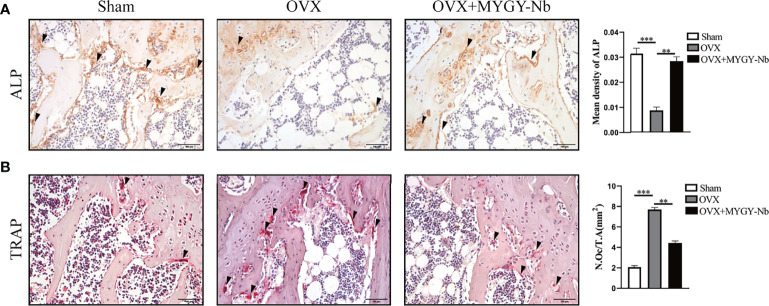
MYGY-Nb treatment restores ALP and TRAP activity in OVX mice. **(A)** ALP immunohistochemical staining and quantification. **(B)** TRAP histochemical staining and quantification. Scale bars: 100 μm. Data were presented as means ± S.D. **P < 0.01; ***P < 0.001. n = 6 in each group.

Studies have shown that the main cause of PMOP is the disorder of bone metabolism contributed by an imbalance between osteoblastic bone formation and osteoclastic bone resorption (34). To determine whether MYGY-Nb regulates the formation of osteoblasts and osteoclasts, we performed TRAP histochemical staining and immunohistochemical staining of ALP. The histological results displayed that MYGY-Nb significantly suppressed OVX-induced increase in TRAP activities and decrease in ALP activities (**Figures 4A, B**), implying that MYGY-Nb prevents OVX-induced bone loss by regulating the formation of osteoclast and osteoblast. Surprisingly, these results are consistent with the effects of some compounds identified by UHPLC-Q-TOF/MS analysis.

### MYGY-Nb Inhibits the Activation of RANKL- Mediated NF-κB Pathway in OVX Mice

**Figure 5:**
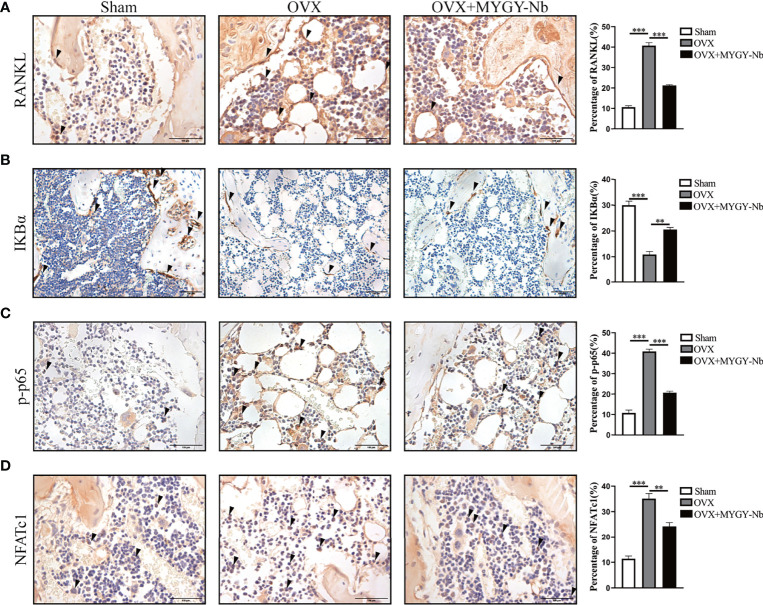
MYGY-Nb inhibited osteoclast formation by suppressing the RANKL-mediated NF-κB pathway in OVX mice. Representative IHC images and quantifications of RANKL **(A)**, IKBα **(B)**, p-p65 **(C)** and NFATc1 **(D)**. Scale bars: 100 μm. Data were presented as means ± S.D. **P < 0.01; ***P < 0.001. n = 6 in each group.

Given that RANKL-induced NF-κB activation is required for osteoclast differentiation and function, we further investigated whether MYGY-Nb inhibits osteoclast formation through the RANKL-mediated NF-κB pathway in OVX-induced mice. By using IHC assay, we found that OVX mice showed a noticeable increase in expression of RANKL, p-p65 and NFATc1, and decrease in expression of IKBα. Nevertheless, after the administration of MYGY-Nb for 12 weeks, the changes of expression of these signaling molecules was inhibited (**Figures 5A–D**). Altogether, these data imply that MYGY-Nb might exert antiosteoporosis effects through the inhibition of RANKL-mediated NF-κB pathway.

### MYGY-Nb Suppresses RANKL-Induced Osteoclast Formation *via* the Inhibition of NF-κB Pathway

**Figure 6:**
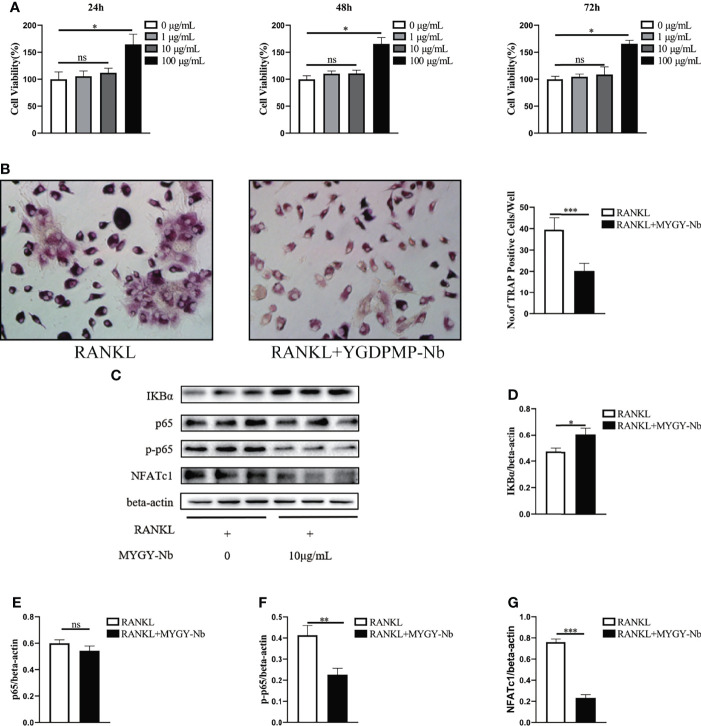
The effect of MYGY-Nb on RANKL-induced osteoclast formation. **(A)** Cell viability of BMMs cultured with various concentrations of MYGY-Nb for 24 h, 48h and 72 h. **(B)** Formation and quantification of TRAP-positive cells from BMMs. **(C–G)** Effects of MYGY-Nb on the activation of the NF-κB signaling pathway in RANKL-induced BMMs. Data are presented as mean ± S.D. (n = 3), *P < 0.05; **P < 0.01; ***P < 0.001; ns, no significant difference, as compared to group treated with RANKL, n = 3 in each group.

To validate the importance of MYGY-Nb in inhibition of osteoclast formation, we performed *in vitro* osteoclast differentiation using BMMs as osteoclast precursors. A CCK-8 assay was conducted to examine the viability of BMMs treated with MYGY-Nb. After treatment for 24, 48 and 72 h, MYGY-Nb showed no effects on BMMs at a dose of 1 and 10 μg/ml, whereas 100 μg/ml MYGY-Nb promoted cell proliferation (**Figure 6A**). Here, we used 10 μg/mL MYGY-Nb to treat BMMs in following osteoclastogenesis assays. After BMMs stimulated by RANKL with or without MYGY-Nb for 6 days, TRAP staining showed that MYGY-Nb led to an inhibition of RANKL-induced osteoclastogenesis (**Figure 6B**). Additionally, we detected the activity of NF-κB signaling pathway. The results indicated that MYGY-Nb significantly suppressed the RANKL-induced degradation of IκBα as well as upregulation of p-p65 and NFATc1 (**Figures 6C–G**). Together, these results indicate that MYGY-Nb inhibits RANKL-induced osteoclast formation by regulating NF-κB pathway.

## Discussion

In present study, we confirmed that MYGY-Nb is the optimal compound combination against PMOP. In OVX mice, MYGY-Nb effectively inhibits bone loss and osteoclastogenesis, as well as decreases the expression of RNAKL and NF-κB signaling genes. Subsequently, *in vitro* experiments further demonstrated that MYGY-Nb suppresses RANKL-induced osteoclast formation through the down-regulation of NF-κB signaling pathway. Altogether, these findings suggest that MYGY-Nb could target RANKL-mediated NF-κB pathway to attenuate osteoclast formation and bone loss.

PMOP is a most common primary osteoporosis (35). However, drugs commonly prescribed for treatment of PMOP have certain side effects, which limit their long-term use. TCM usually consists of several herbs that interact with each other to produce synergistic effects, which not only improve the efficacy but also may reduce the toxic side effects produced by single drugs (36, 37). It is well known that proper extraction of TCM formulas can optimize the synergy between effective compounds to avoid the overdose of ineffective compounds (38–40). MYGY is refined from the classic formula for kidney tonic, You-Gui-Yin, and has great anti-osteoporosis efficacy. In this study, we used N-butanol to extract MYGY and then explored its efficacy in OVX-induced osteoporosis mice. Surprisingly, ABH staining and μCT analysis both showed that MYGY-Nb was more effective in the inhibition of bone loss than the original MYGY formula, implying that the combination of compounds in MYGY-Nb has better therapeutic effects on PMOP.

In the pathogenesis of PMOP, over-activated osteoclast formation and excessive bone resorption are significant factors (23). Thus, the inhibition of osteoclast formation is an important strategy for PMOP treatment (41). In the present study, our data showed that MYGY-Nb significantly decreased the number of osteoclasts in OVX mice, which indicated that MYGY-Nb might regulate the process of osteoclastogenesis. The *in vitro* experiments further confirmed that MYGY-Nb significantly prevented RANKL-induced BMMs differentiation and suppressed the osteoclast formation. Then, we sought to clarify the combination of compounds in MYGY-Nb. UPLC/MS identified 16 major compounds of MYGY-Nb, in which kynylpinoside, rutinoside (Rutinos)_1, glycyrrhizin and glycyrrhetinic acid have been reported to have inhibitory effects on osteoclast formation (25–28). Our future studies are warranted to investigate the anti-osteoporosis efficacy of these compounds in more detail.

RANKL signaling is thought to be the major target of anti-resorptive agents that inhibit bone loss and osteoclast activation. RANKL-induced osteoclast differentiation activates NF-κB and NFATc1 (42–44). Under normal conditions, NF-κB protein is stored as an inactive form in the cytoplasm (the p50/p65 heterodimer associated with repressive IκBα) (45). Upon stimulation by RANKL, IκBα undergoes phosphorylation resulting in the masking of the nuclear localization signal on p65, which allows NF-κB to translocate to the nucleus. Previous studies have shown that the NF-κB inhibitor(-)-dehydroxymethylepoxyquinomycin blocks RANKL-induced osteoclast formation by down-regulating NFATc1 expression (46–49). In the present study, we found that MYGY-Nb suppressed OVX-induced expression of RANKL, p-p65 and NFATc1. In addition, MYGY-Nb also decreased the expression of IκBα, p-p65 and NFATc1 in RANKL-treated BMMs. Collectively, these data indicated that MYGY-Nb might play a key role in regulation of RANKL-mediated NF-κB signaling pathway. Thus, more precise genes of the NF-κB signaling pathway targeted by MYGY-Nb need to be explored in future studies.

## Conclusions

In conclusion, our data reveal that MYGY-Nb exerts anti-osteoporotic efficacy by inhibiting osteoclast formation and reducing bone loss. Furthermore, we validated that the RANKL-mediated NF-κB signaling pathway plays an important role in the anti-osteoporosis efficacy of MYGY-Nb. Our findings provide evidence that MYGY-Nb is a promising therapeutic drug for PMOP.

## Data Availability Statement

The original contributions presented in the study are included in the article/supplementary material. Further inquiries can be directed to the corresponding authors.

## Ethics Statement

The animal study was reviewed and approved by Animal Ethics Committee of Zhejiang Chinese Medical University.

## Author Contributions

Study conception and design: QZ, RX, and JC. Data acquisition: HL, WY, XW, SZ, MG, and PW. Data analysis and interpretation: XW, SZ, TX, HR, and HJ. Article drafting: QZ, RX, and HL. Critically revising important intellectual content: HQ, FY, and JC. Final approval of the version of the article to be published: HQ, FY, and JC.

## Funding

This research has been partially supported by the Natural Science Foundation of China (Grant nos. 81904223), Hangzhou Medical and health science and Technology Project (B20200552).

## Conflict of Interest

The authors declare that the research was conducted in the absence of any commercial or financial relationships that could be construed as a potential conflict of interest.

## Publisher’s Note

All claims expressed in this article are solely those of the authors and do not necessarily represent those of their affiliated organizations, or those of the publisher, the editors and the reviewers. Any product that may be evaluated in this article, or claim that may be made by its manufacturer, is not guaranteed or endorsed by the publisher.
